# Neonatal Compartment Syndrome

**Published:** 2015-10-28

**Authors:** Sagar Mehta, Jayant Agarwal

**Affiliations:** University of Utah School of Medicine, Salt Lake City

**Keywords:** compartment syndrome, upper extremity ischemia, neonatal compartment syndrome, in utero extremity insult, extremity birth deformity

**Figure:**
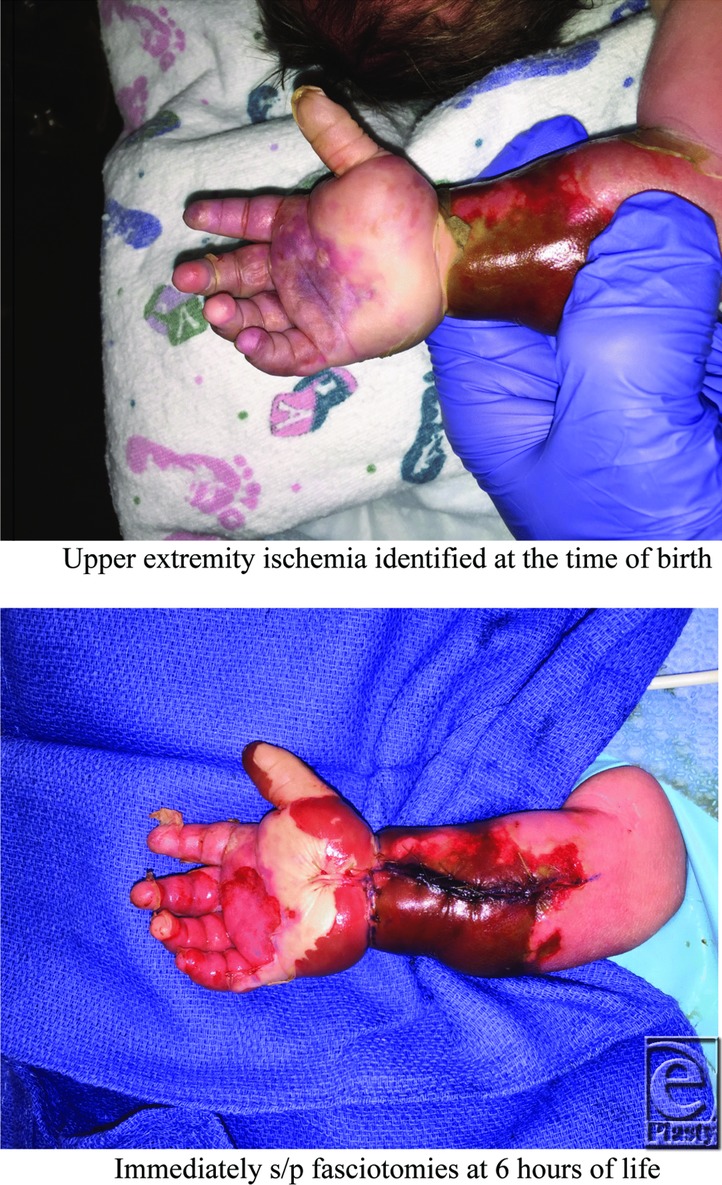


**Figure:**
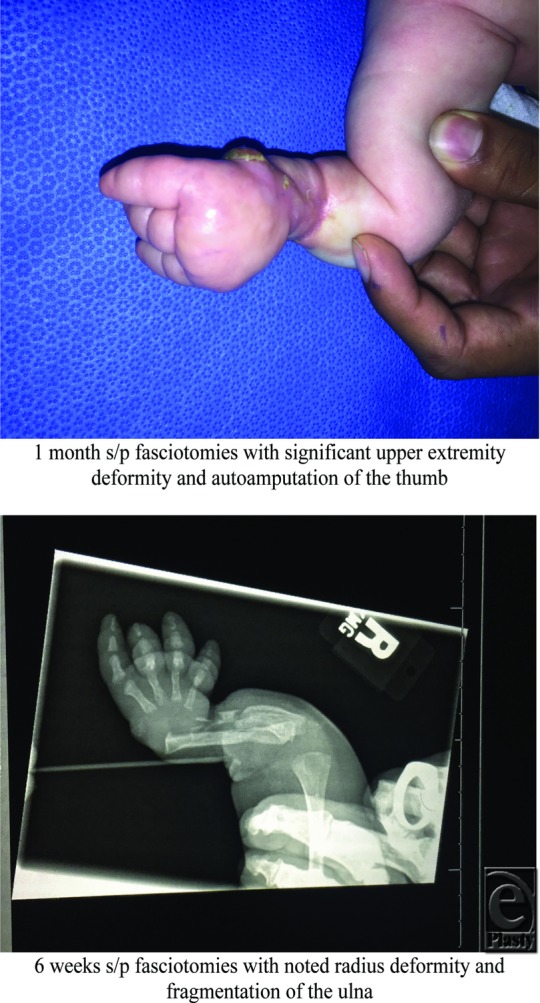


## DESCRIPTION

A 7-lb, 39-week-old newborn was born to a 45-year-old G14P8238 woman by cesarean delivery for prolonged rupture of membranes. The patient presented with desquamation of the palm and a leathery appearance of the skin to the right dorsal hand and circumferential forearm with frank ischemia to the thumb.

## QUESTIONS

1. **What are the causes of neonatal compartment syndrome?**
2. **What factors (maternal or neonatal) correlate with neonatal compartment syndrome?**
3. **What are the signs and symptoms of neonatal compartment syndrome?**
4. **What options are available for the treatment of neonatal compartment syndrome?**

## DISCUSSION

The cause of neonatal compartment syndrome is often unclear. The literature differentiates the causes for compartment syndrome as either extrinsic or intrinsic. Extrinsic causes include oligohydramnios, umbilical loops, or amniotic bands. Intrinsic causes include arterial embolus and neonatal hypercoagulability.^1^^,^^2^ When attempting to determine the cause of compartment syndrome, it is important to assess factors associated with the mother as well as with the neonate. In the described case, it is possible that advanced maternal age and/or arterial thromboembolism were associated with the upper extremity ischemia.

In a case series of 24 patients published by Ragland et al,^2^ there were no specific neonatal or maternal conditions that had a strong correlation with compartment syndrome. A study that assessed 50 cases of neonatal ischemic contracture found that approximately 20% of the population was preterm.^1^ Many factors have been assessed including maternal/neonatal infections, prematurity, difficulty of labor, maternal diabetes, and oligohydramnios. There is currently no clear consensus for the cause of neonatal compartment syndrome, and no single factor has a strong correlation with neonatal compartment syndrome.

In a majority of reported cases of neonatal compartment syndrome, some degree of skin desquamation was noted on the arm as a heralding sign.^1^^,^^2^ However, there was a gradation of symptoms in patients. Reported symptoms ranged from skin and skeletal involvement to flexor or extensor compartment ischemia to both flexor and extensor ischemia with or without skeletal involvement to late contractures.^2^ Interestingly, no patients in the literature were noted to have systemic signs of compartment syndrome including rhabdomyolysis or renal failure. This is possibly secondary to maternal clearance of toxins.

Treatment options for these patients are often limited and are dependent on the degree of ischemia. A decision to emergently go to the operating room is often clouded by the unclear clinical picture. The risk of anesthesia in newborns is significant, with neonates less than 30 days old having a significantly higher mortality rate than older children.^3^ In one large series of neonatal forearm ischemia, patients underwent delayed surgical revision rather than immediate exploration and fasciotomy.^1^ The type of revision surgical procedures varied on the basis of degree of ischemia and included revision of skin necrosis or scarring, tendon transfers for correction of severe compartment contracture, or amputation of the most severely affected structures. A small number of patients reported in the literature underwent emergent surgery for hand or forearm ischemia, which resulted in successful salvage in the extremity.^4^^,^^5^ There are also 2 reported cases describing the early use of urokinase, a thrombolytic agent, for suspected arterial thrombosis.^6^

Amputation in the neonate should be carefully planned. Important goals when considering amputation include preservation of limb length and maintaining good soft-tissue coverage over the bony portions of the extremity. Adequate length and soft-tissue durability are necessary for potential future prosthesis fitting and use. If the neonate undergoes amputation too early, he or she may not have sufficient soft-tissue coverage, which can limit the ability to preserve adequate length. This was an important consideration in our patient who will likely undergo delayed amputation in hopes of preserving soft-tissue coverage and limb length.

To some extent, neonatal compartment syndrome continues to remain an enigma. Because of the low incidence and limited discussion in the literature, there is not a clear understanding of causation for the event or correlating factors. This syndrome displays a varied clinical presentation and therefore treatment plans for each neonate should be individualized.

## References

[1] Agrawal H, Dokania G, Wu SY (2014). Neonatal Volkmann ischemic contracture: case report and review of literature. AJP Rep.

[2] Ragland R, Moukoko D, Ezaki M, Carter PR, Mills J (2005). Forearm compartment syndrome in the newborn: report of 24 cases. J Hand Surg Am.

[3] van der Griend BF, Lister NA, McKenzie IM (2011). Postoperative mortality in children after 101,885 anesthetics at a tertiary pediatric hospital. Anesth Analg.

[4] Plancq MC, Buisson P, Deroussen F, Krim G, Collet LM, Gouron R (2013). Successful early surgical treatment in neonatal compartment syndrome: case report. J Hand Surg Am.

[5] Isik C, Demirhan A, Karabekmez FE, Tekelioglu UY, Altunhan H, Ozlu T (2012). Forearm compartment syndrome owing to being stuck in the birth canal: a case report. J Pediatr Surg.

[6] Ricciardelli E, Morgan RF, Lin KY (1995). In utero brachial artery thrombosis: limb salvage with postnatal urokinase infusion. Ann Plast Surg.

